# Risk factors for orofacial clefts in India: A case–control study

**DOI:** 10.1002/bdr2.1073

**Published:** 2017-08-02

**Authors:** Sutapa Bandyopadhyay Neogi, Samiksha Singh, Dinesh Raj Pallepogula, Hira Pant, Sunanda Reddy Kolli, Priyanka Bharti, Vikram Datta, Srinivas Reddy Gosla, Krishnamurthy Bonanthaya, Andy Ness, Sanjay Kinra, Pat Doyle, Venkata Satyanarayana Murthy Gudlavalleti

**Affiliations:** ^1^ Indian Institute of Public Health‐ Delhi Public Health Foundation of India New Delhi India; ^2^ Indian Institute of Public Health‐ Hyderabad Public Health Foundation of India Hyderabad India; ^3^ Centre for Applied Research and Education on Neurodevelopmental Impairments and Disability related Health Initiatives (CARENIDHI) New Delhi India; ^4^ Kalawati Saran Hospital and Lady Hardinge Medical College New Delhi India; ^5^ GSR Institute of craniofacial surgery Hyderabad India; ^6^ Bhagwan Mahaveer Jain Hospital Bengaluru India; ^7^ The UK National Institute for Health Research Bristol Nutrition Biomedical Research Unit University of Bristol Bristol United Kingdom; ^8^ London School of Hygiene and Tropical Medicine London United Kingdom

**Keywords:** Orofacial clefts, cleft lip/palate, risk factors, folic acid supplementation

## Abstract

**Background:**

Orofacial clefts (OFC) are linked with several genetic and environmental factors. The aim of this study was to explore the association of potential risk factors with OFCs in India.

**Methods:**

This was a hospital‐based, matched case–control (1:4 ratio; matching done for parity) study conducted in Hyderabad, Bengaluru, and Delhi‐National Capital Region. Cases (nonsyndromic clefts) were recruited from treatment centers, while controls (live births) were recruited from maternity centers. Information on exposures was collected during personal interviews. Exposures of interest included folic acid supplementation during the peri‐conceptional period, consanguineous marriage, exposure to drugs, infections during pregnancy, family history of OFC, and dietary factors.

**Results:**

A total of 785 participants were included in the study: 157 cases and 628 controls. A family history of cleft lip/palate (adjusted odds ratio [AOR], 15.48; 95% confidence interval [CI], 4.36–54.96; *p* value = 0.001), exclusive vegetarianism (AOR, 4.47; 95% CI, 1.83–10.98; *p* value = 0.001), and delayed first conception (AOR, 2.55, 95% CI, 1.25–5.21, *p* = 0.01) were found to be strongly associated with higher risk of OFCs. Supplementation with folic acid during first 3 months of pregnancy was not found to be protective against OFCs (AOR, 1.24; 95% CI, 0.59–2.58; *p* value = 0.56).

**Conclusion:**

Our study confirmed the importance of family history as a risk factor for OFC. Our study did not show an association with folic acid supplementation but was underpowered to detect small effects. Our finding of higher risk among vegetarians requires replication. Birth Defects Research 109:1284–1291, 2017. © 2017 Wiley Periodicals, Inc.

## Introduction

The burden of orofacial clefts (OFC) is a major concern in India and throughout the world (Mossey et al., 2009). OFC includes cleft palate (CP) and cleft lip (CL) in isolation or in combination. Children suffering from OFCs in low income countries have high morbidity throughout their life. They face a range of functional and cosmetic problems with potential long‐term adverse impact on health, speech, hearing, psychology, learning ability and social skills (Agbenorku, 2013). The treatment often requires a multidisciplinary approach, from surgical correction of physical defect to long‐term psychological counselling (Mossey and Little, 2009).

Estimates from a meta‐analysis including 11 hospital based studies reported a prevalence of OFCs ranging from 0.2 to 2.9 per 1000 total births and a pooled prevalence of 1.3 per 1000 total births in India (Allagh et al., 2015). It is estimated that every year 28,600 infants are born with cleft lip and cleft palate, that is, 78 infants per day in the country (Mossey and Little, 2009).

The etiology of OFC is polygenic and multi‐factorial. Both genetic and environmental factors have been identified though the precise cause in many cases is unclear (Mossey and Little, 2009). The environmental factors include maternal illness, infections, drugs, radiation, alcohol, and contamination of food and water with pesticides, nitrates, and mercury (Aylsworth et al., 2015). In vitro fertilization and intracytoplasmic sperm injection, consanguinity, dietary, and indigenous medicines used for sex selection are also implicated as risk factors (Mossey and Little, 2009; Neogi et al., 2015).

Folic acid deficiency has been linked to the causation of many chronic and developmental disorders (Smithells et al., 1976; Botto et al., 1999; Caudill M, 2008; Molloy et al., 2008, 2009). Folate deficiency is prevalent globally, especially in low and middle income countries, ranging between 20 and 30% (Gamble et al., 2005; Pathak et al., 2007; McLean et al., 2008). Various studies have suggested a protective effect of peri‐conceptional folic acid consumption from cleft lip with or without palate. However, the evidence is inconsistent. This is further compounded by heterogeneity resulting from different dosages of folic acid and types (with or without other multivitamins) (De‐Regil et al., 2010).

Most of these studies on risk factors and prevention of OFCs by folic acid supplementation were done in Western countries (Figueiredo et al., 2015). Comparatively, India has a predominantly vegetarian population, low maternal vitamin‐B12 status, and higher prevalence of consanguineous marriages, infections, and maternal illnesses. Thus, the risk profile of OFCs may be different (Agbenorku, 2013). Therefore, the aim of our study was to assess risk factors of OFCs, including lack of folic acid supplementation, during peri‐conceptional period.

## Materials and Methods

### Study Design and Settings

This was a hospital‐based, matched case–control (1:4 ratio; matching for parity) study conducted in Hyderabad, Bengaluru, and Delhi. At each site, cases were selected from a treatment center specialized for managing OFCs. These treatment centers were part of Smile Train, a program run by a nongovernmental organization having a nationwide chain of accredited centers that provide free treatment for OFCs. The cases were recruited from the following treatment centers: GSR Institute of Craniofacial Surgery, Hyderabad; Bhagvan Mahaveer Jain Hospital, Bangalore, Karnataka; Kalawati Saran Children's hospital, Delhi; Sant Paramand Hospital, Delhi. For controls, the following centers were selected: Koti Maternity Hospital, Mahbubnagar; District Hospital Mahbubnagar District, Telangana; Kalawati Saran Children's Hospital, Delhi; Srirampura referral Hospital, Bangalore; Bhanshankari District Hospital, Bangalore; Ulsoor referral hospital, Bangalore; Siddipur referral Hospital, Bangalore. Controls were selected from maternity centers near/adjoining the treatment centers. Women who delivered in these centers during the study period were recruited after they gave their consent.

The study population consisted of all pregnant women delivering in these hospitals or visiting treatment centers for their children with cleft lip/ palate who gave their consent to participate. When selecting controls, we excluded stillbirths or babies who had any apparent structural or chromosomal malformations other than OFC, if they were part of any other on‐going studies, had any maternal complications that warranted emergency care, babies referred from other hospitals or who were not from the catchment area, or those not willing to participate in the study.

### Cases and Controls

#### Cases

Any baby with nonsyndromic clefts, that is, cleft lip ± palate or cleft palate, who visited the treatment center within 4 months of birth was considered as a case. The treating physician ascertained the outcomes based on the standard case definitions used to detect OFCs. OFCs in this study included an opening in a structure around the mouth and face, including clefts in the lip, the roof of the mouth (hard palate), or the tissue in the back of the mouth (soft palate). We interviewed the mothers of the cases at the time of recruitment.

#### Controls

The controls were live births without any malformation born in a maternity center close to the same region as the treatment center of the case. We interviewed mothers of the controls within 48 hours of delivery. We verified that both cases and controls were inhabitants of the same geographical regions from their addresses, and close to the sites where the study was conducted. Each control selected purposively was individually matched to her control according to parity of mother, using the groups: primiparous, parity 1, 2, and 3 +. The study aimed to match four controls to each case.

### Sample Size

For each case, four controls were selected. The estimated sample size calculated using Open Epi software (version 3.1) (Dean et al., 2013) was 750 (150 cases and 600 controls) assuming an alpha error of 10%, a power of 80% to detect a 50% decrease in risk (odds ratio [OR] = 0.5) associated with folic acid supplementation. The prevalence of folic acid supplementation in the control group was assumed to be around 20% with 5% nonresponse rate.

### Data Collection and Management

Data collection took place between January 2015 and July 2016. Structured data collection questionnaires developed by the research team captured information on basic socio‐demographic features, details of reproductive, medical and occupational history and other potential risk factors. We translated the tools into local languages, pretested, piloted, and modified before data collection.

Trained female field investigators interviewed mothers to collect information on risk factors. Ascertainment of exposures was based on the history elicited using a structured questionnaire designed for the study. Data on folic acid supplementation were collected based on the history of intake of folic acid tablets during the peri‐conceptional period and dietary history.

We collected data on paper forms. Data entry was done by staff trained by the data manager using customized forms designed and validated in MS Access with built in consistency, range, and missing data checks by the data base administrator.

### Exposures

The risk factors included in the study were lack of folic acid supplementation during peri‐conceptional period along with other documented risk factors, which were conception induced by medications or treatment; exposure to radiation (x‐rays); infections; medical illness during pregnancy (such as epilepsy, hypertension, diabetes mellitus, fever, rash, arthritis, common cold, urinary tract infection, and vomiting); intake of any medicinal drugs; food habits (vegetarian/nonvegetarian); consumption of coffee, alcohol, tobacco, toddy (local alcoholic drink) or any drugs; exposure to smoking, indoor pollution, pesticides during the peri‐conceptional period; consanguineous marriage; family history of any malformations; increased maternal and paternal age (Jha, 2001; Agbenorku, 2013; Neogi et al., 2015). Information was collected by interviewing the women as well as by reviewing medical records wherever feasible.

### Ethical Consideration

We obtained informed consent from eligible mothers after giving them detailed information about the study in local language. We sought approvals from the respective hospitals and Institutional Ethics committee of Public Health Foundation of India.

### Statistical Analysis

STATA 11.0 was used for analysis. The socioeconomic characteristics of cases and controls were compared using the unpaired *t* test for continuous variables and the Chi‐square test for categorical variables. Associations between exposures and outcomes were assessed for case–control pairs using unadjusted and adjusted conditional logistic regression. For the adjusted analysis, exposures that had different distributions in cases and controls (*p* < 0.05) (and with at least five respondents in each category), and those having a biological plausibility as potential confounders (irrespective of *p* value) were included in the adjusted conditional logistic regression model.

## Results

A total of 785 participants were included in the study: 260 from north India (Delhi) and the rest from south India (250 from Hyderabad and 275 from Bengaluru). There were no refusals. Nearly equal proportion of males and females were present in the study group, and there was nearly equal distribution (48.4%) of primiparous and multigravida (51.6%) mothers. Most of the mothers were literate. Most households (452; 57.6%) belonged to backward castes, and 63.8% had family income less than one lakh Indian rupees (1500 USD) per year. A large proportion (86%) of women were homemakers. Approximately 7% of fathers worked in agriculture. The mean age at marriage for mothers was 20.2 years (SD, 3.1), ranging from 10 to 37 years; 16.4% of the overall study population reported consanguinity or marriage with relatives, with Delhi (20%) reporting the maximum (Supplementary Table S1, which is available online).

### Socio‐demographic Characteristics of Cases and Controls

The study had 157 matched case–control pairs (1:4). The cases and controls were similar with respect to most of the socio‐demographic variables. In both the categories, the majority of mothers did not have employment outside the home and most fathers worked in office jobs, as manual laborers or were in business. The majority among cases and controls belonged to backward castes, and most cases and controls had an annual family income less than one lakh Indian rupees (Table 1).

**Table 1:** Socio‐demographic Profile of Cases and Controls

Variables	Cases (*n* = 157)	Control (*n* = 628)	*p*‐Value
Age of child in days [mean (SD)]	61.9 (39.1)	14.4 (29.1)	0.000
Age of the child more than or equal to 14 days at time of interview [*n* (%)]	133 (84.7)	114 (18.2)	0.000
Sex of child ‐ male [*n* (%)]	64 (40.7)	297 (47.3	0.14
Age of mother (in completed years) [mean (SD)]	24.8 (3.8)	24.0 (3.5)	0.01
Age of father (in completed years) [mean (SD)]	29.7 (4.3)	28.8(4.3)	0.02
Mothers less than 30 years of age	138(87.9)	575 (91.5)	0.15
Fathers less than 30 years of age	80 (50.9)	368 (58.6)	0.08
Education status of mother – illiterate or just literate [*n* (%)]	18 (11.4)	78 (12.4)	0.74
Education status of father‐ illiterate or just literate [*n* (%)]	14 (8.8)	90 (14.3)	0.07
Occupation of mother ‐ homemaker [*n* (%)]	134 (85.4)	542 (86.3)	0.76
Occupation of father‐ agriculture/ industry [*n* (%)]	25 (15.9)	77 (12.2)	0.24
Caste backward caste [*n* (%)]	74 (47.1)	378 (60.1)	0.003
Family income less than 1 lakh rupees per year [*n* (%)]	110 (98.2)	391 (99.7)	0.09
Age at marriage [mean (SD)]	20.5 (3.3)	20.1 (3.0)	0.2
Drinking water (ground water) [*n* (%)]	41 (26.1)	130 (20.7)	0.14
Fuel used in cooking‐ kerosene/ biomass/ coal [*n* (%)]	18 (11.5)	89 (14.2)	0.08
Place of cooking inside the house [*n* (%)]	146 (93)	583 (92.8)	0.6
No. of living children [mean (SD)]	1.7 (0.07)	1.6 (0.03)	0.07
H/o of consanguineous marriage	28 (17.8)	101 (16.1)	0.6
H/o of abortion	47 (30.0)	131 (20.8)	0.01
H/o neonatal death	5 (3.2)	18 (2.9)	0.83
H/o stillbirths	0	6 (1.0%)	
Current multiple pregnancy	5 (3.2)	6 (0.9)	0.03
H/o multiple pregnancy in previous pregnancies	3 (1.9)	1(0.2)	0.00
H/o previous child with cleft lip	3 (1.9)	0	
Any family history of cleft lip or palate	27 (17.2)	10 (1.6)	0.000
Any exposure to pesticide/ herbicides	56 (35.7)	235 (37.4)	0.6
More than 1 year for conception	106 (67.5)	324 (51.6)	0.000
H/o any treatment taken for infertility	24 (15.3)	32 (5.1)	0.00
Medical illnesses in the first 3 months of pregnancy			
– Fever	23 (14.6)	85 (13.5)	0.71
– Rash	11 (7.0)	17 (2.7)	0.01
– Hypertension	6 (3.8)	15 (2.3)	0.32
– Epilepsy	2 (1.3)	3 (0.5)	0.263
– Arthritis/ chikungunya	1 (0.6)	8 (1.3)	0.50
– Urinary tract Infection	25 (15.9)	65 (10.3)	0.05
– Diabetes	1 (0.6)	2 (0.3)	0.56
H/o intake of medicine in first 3 months for any illness	76 (48.4)	276 (43.9)	0.3
H/o consumption folic acid			
– 3 months before pregnancy	9 (5.7)	4 (0.6)	0.00
– First 3 months of pregnancy	48 (30.6)	136 (21.6)	0.02
H/o consumption of iron			
– 3 months before pregnancy	7 (4.4)	5 (0.8)	0.001
– 3 months of pregnancy	70 (44.6)	181 (28.8)	
H/o consumption of multivitamin during peri‐conceptional period	47 (29.9)	134 (21.3)	0.02
H/o X ray in first 3 months of pregnancy	5 (3.2)	5 (0.8)	0.03
Food habit‐vegetarianism	46 (29.3)	72 (11.5)	0.00
Exposure to passive smoking in peri‐conceptional period	47 (29.9)	110 (17.5)	0.60
Exposure to active smoking	1 (0.6)	1 (0.2)	0.30
H/o chewing tobacco in peri‐conceptional period	1 (0.6)	4 (0.6)	0.31
H/o consumption of any form of alcohol in periconceptional period	5 (3.2)	38 (6.0)	0.10

#### Association between exposures and OFC

A total of 13 women out of 785 reported consuming folic acid during 3 months preceding conception. However, more mothers of cases reported taking folic acid (30.6%) compared with controls (21.6%) during first 3 months of pregnancy. Almost one‐third of the mothers of cases were vegetarians compared to 11% of mothers of controls.

Among the exposures examined, history of previous abortion, family history of OFC, more than 1 year taken for conception, history of any treatment taken for infertility, history of rash, urinary tract infection in first 3 months of pregnancy, history of consumption of folic acid or iron and folic acid in first 3 months of pregnancy, multivitamin consumption, vegetarianism, backward caste, and age of the child at the time of interview were found to be associated (*p* < 0.05 in unadjusted analysis) with OFCs (Table 2).

**Table 2:** Association between Exposures and OFC

Factors^a^	Unadjusted model		Final model	
	OR	P value	AOR	*p*‐Value
H/o consumption folic acid in first 3 months of pregnancy	1.61 (1.07‐2.41)	0.02	1.24(0.59‐2.58)	0.56
H/o consumption of iron in first 3 months of pregnancy	4.80 (1.46‐15.73)	0.01	3.29 (0.45‐ 23.84)	0.24
Food habit ‐vegetarianism	3.73 (2.32‐5.99)	0.000	4.47 (1.83‐ 10.98)	0.001
Age of the child more than or equal to 14 days at time of interview	31.06(17.08‐ 56.52)	0.000	34.29 (16.70‐ 70.42)	0.000
Maternal age more than or equal to 30 years	0.87 (0.47‐1.64)	0.68	0.86 (0.29‐2.47)	0.77
Backward caste	2.01 (1.34‐3.03)	0.001	1.92 (1.01‐ 3.68)	0.04
H/o of abortion	1.61 (1.06‐2.43)	0.02	1.62 (0.75‐3.52)	0.22
Current multiple pregnancy	3.33 (1.02‐10.92)	0.05	0.6 3(0.07‐ 6.07)	0.69
Any family history of cleft lip or palate	12.93 (5.86‐28.54)	0.000	15.48 (4.36‐ 54.96)	0.000
More than 1 year for conception	2.10 (1.42‐3.12)	0.0001	2.55 (1.25‐ 5.21)	0.01
H/o any treatment taken for infertility	3.67 (2.05‐6.58)	0.000	1.15 (0.42‐3.19)	0.78
Medical illnesses in the first 3 months of pregnancy				
– Rash	2.58 (1.21‐5.52)	0.01	3.37 (0.84‐13.49)	0.09
– Urinary Tract Infection	1.58 (0.94‐2.65)	0.08	1.22 (0.43‐3.47)	0.71
H/o X ray in first 3 months of pregnancy	4.93 (1.32‐18.35)	0.02	10.66 (1.03‐ 110.10)	0.05

a Factors included are those that differed significantly between cases and controls (*p* < 0.05) (and having at least five respondents in each category), and those having a biological plausibility as potential confounders (irrespective of the *p* value).

A model of multivariable analysis was developed using conditional logistic regression (Table 2). In the final model, a family history of cleft lip/palate, exclusive vegetarianism and delayed first conception beyond one year were associated with OFCs. Cases had 15 times higher likelihood of having a family history than controls (adjusted odds ratio [AOR], 15.5; 95% confidence interval [CI], 4.4–54.9). Likewise, cases were around 4.5 times more likely than controls to be exclusive vegetarians (AOR, 4.5; 95% CI, 1.8–10.9). Cases were more likely to be born after a delayed conception compared with controls (AOR, 2.55; 95% CI, 1.25–5.21). Supplementation of folic acid 3 months before conception had to be excluded from the adjusted analysis owing to small cell sizes. Supplementation for 3 months after conception did not seem to have a protective effect in the final adjusted analysis (AOR, 1.2; 95% CI, 0.6–2.6). Age of child emerged as a strong factor (AOR, 34.3; 95% CI, 16.7–70.4), reflecting the fact that cases were older at the time of interview than the controls.

## Discussion

In this case–control study of OFCs, we found that a family history of cleft lip/palate, vegetarianism, and delayed first conception beyond 1 year were associated with a higher risk of OFCs. We did not find any evidence to support a protective association with folic acid supplementation during first 3 months of pregnancy.

A family history of OFC is a strong risk factor for nonsyndromic OFCs as shown in previous studies (Acuna‐Gonzalez et al., 2011; Rahimov et al., 2012; Burg et al., 2016). In our study, the strength of association was as high as 15 but with very wide CIs. Our findings are consistent with other studies (effect size varying from 17 to 56) (Sivertsen et al., 2008; Grosen et al., 2010). Delayed first conception beyond 1 year emerged as a risk factor in our analysis. Treatment received for infertility was associated with OFCs in unadjusted analysis but did not show any association in the adjusted analysis.

In the analysis, supplementation with multivitamin or folic acid preparations in the peri‐conceptional period did not seem to have any protective effect. However, these findings should be interpreted in the Indian context where any preparations consumed for “strength and vitality” are often termed as “multivitamin preparations” by the people. So, self‐reported consumption of multivitamins could include either multivitamins, folic acid, iron preparations, or indigenous medicines as well.

It is also important to note that in India supplementation usually starts only after the first antenatal visit, which usually takes place after the missed period or approximately 3 to 4 weeks after conception. The fetal lip usually closes by 5 to 6 weeks after conception and the palate by 10 weeks. This gives a narrow or no window of opportunity for peri‐conceptional folic acid supplementation to be administered and to be effective. The reported intake of supplementation in the pre‐conceptional period was low (5.7% among mothers of cases and 0.6% among controls). Reported intake during the first 3 months of pregnancy was higher, 30.6% among cases and 21.6% among controls, but we have no details on when they initiated supplementation.

Previous studies on the association of multivitamins with OFCs have reported mixed findings, although multivitamin preparations containing folic acid have suggested a protective effect (Czeizel, 1993; Bailey and Berry, 2005). The independent role of folic acid is still unclear (Shaw et al., 1995; Itikala et al., 2001). Lack of preventive effect by folic acid supplementation was reported in some studies with a large sample size of over 3000 (Hayes et al., 1996; Czeizelet al., 1999, 2004). Other studies suggested that folic acid was effective only at higher doses (6–8 mg) (Czeizel et al., 1999). One study suggested efficacy of multivitamin and folic acid supplementation in unilateral cleft with a 83% risk reduction, while no effect was observed for bilateral cleft (Tolarova and Harris, 1995). A Cochrane review conducted in 2010 suggests that there is no evidence of any effect of folic acid supplementation on prevention of cleft palate and/or cleft lip (De‐Regil et al., 2010). Yet another study suggests that fortification with folic acid may create an imbalance between folic acid and vitamin‐B12, further aggravating the risk of having a child with OFCs (Godbole et al., 2009).

Of interest, vegetarianism as compared to nonvegetarian diet emerged to be strongly associated with OFC in our study. Women having vegetarian diets have low folate and vitamin B12 levels (Kirchheimer et al., 2001; Refsum et al., 2001) and a diet deficient in folate and vitamin B12 was shown to be related with OFC (Krapels et al., 2004). Most vegetarians in India adopt traditional cooking methods with overexposure of food to heat. Fifty to 90% of folate can be destroyed by these traditional cooking methods because of folate acid's sensitivity to heat (Wardlaw, 2004). Moreover, most of them are exclusive vegetarians and, therefore, prone to vitamin B12 deficiency, which is essential for increasing the bioavailability of folate. Vitamin B12 deficiency could increase risk of OFCs as vitamin B12 plays a role in many enzymatic reactions linked to folic acid metabolism (Wardlaw, 2004). A vegetarian diet may also increase exposure to pesticides through agricultural use although the evidence is equivocal. Vegetarian populations may be more exposed to pesticide residues (except for organochlorine compounds) than the general population (Van Audenhaege et al., 2009). Nonvegetarians may also be exposed to concentrated forms of pesticides because animals eat plant products, and fat soluble contaminants or those that are resistant to degradation accumulate in the fat tissues that ultimately reach humans (Dórea, 2004).

One of the limitations of our study was that the data were primarily based on recall. There is generally no documentation or medical records available on the frequency and duration of folic acid or multivitamin supplementation in our settings. Multivitamin preparations are available in diverse formulations, these may or may not contain folic acid, and people tend to interpret supplements as anything that is given for strength and immunity. These were, therefore, excluded from the final analysis. On the other hand, folic acid preparations are uniform and it is relatively easier to elicit information from mothers because of its consistency in color and size. Moreover, when Indian women visit a doctor or a facility in the first trimester of pregnancy, any medicine prescribed during the first visit is less likely to be forgotten. Nonetheless, there could be a problem with recall bias in both cases and controls giving rise to nondifferential misclassification of folic acid status. Moreover, in our study, cases were interviewed when they came for surgical intervention while controls were interviewed 48 hr after the delivery. This could have introduced a selection bias because we selected cases whose parents sought care.

Cases and controls should be chosen from the same base population to reduce selection bias in case–control studies. Evidence shows that for hospital‐based case–control studies, community controls are the best choice if high response rates can be attained (Neupane et al., 2010). However, considering the budget constraints for any public health research in resource‐scarce settings, researchers may consider selecting hospital controls for a case–control study if potential confounders are carefully considered, measured, and adjusted for (Rahman et al., 2012). In our study, although we attempted to select controls from the same geographical regions from their addresses, they might not have represented the population that gave rise to cases. There is, therefore, a possibility of some degree of selection bias in our study.

There were no disaggregated data on the type of OFC; literature suggests that determinants (both environmental and genetic etiologies) of OFC vary with the type (Bianchi et al., 1997; Burg et al., 2016). Combining cleft lip and palate together may have attenuated the strength of association that we would have otherwise obtained. There seems to be a strong association of caste with OFCs. Because these were based only on self‐reports in our study and there is a likelihood of people not revealing the actual caste, we combined all backward castes as a single category and compared it against nonbackward castes in the final analysis. Sociodemographic factors, however, are less amenable to change during the course of pregnancy and, hence, unlikely to be affected by recall or misclassification bias.

On the other hand, factors related to medical conditions are less likely to be missed because every subject was enrolled from the hospitals where these details are recorded routinely. Gathering information from medical records leads to less recall bias. Information on diet is highly subject to bias because it is likely to get altered during the course of pregnancy. So, we restricted ourselves only to analyzing whether the person was vegetarian or nonvegetarian. Vegetarianism is strongly linked with sociocultural customs and generally remains unaltered in Indian culture. The possibility of a vegetarian person getting misclassified as a nonvegetarian is much less, although the converse may be true. However, triangulation was done to overcome this issue by asking mothers about the frequency of intake of nonvegetarian food before and after pregnancy. Given the limitations of recall, we could not categorize participants into those consuming non vegetarian food daily, weekly, or occasionally, and we combined them in one group.

To our knowledge, this is one of only a few case–control studies evaluating risk factors of OFC in a largely vegetarian population. Although, we assumed that there would be a 5% nonresponse rate, in our study there were no refusals which adds to its strength. It was conducted in two geographically and culturally different (north India vs. south India) areas within India. This increased the external validity of the study. Cases were taken based on physician's diagnosis only and all the subjects were recruited from big hospitals where the system of recording is better than many hospitals of the country. Our team reviewed medical records and collected information from them for most of the risk factors studied, minimizing information bias as far as possible. In addition, having four controls for every case improved the power and efficiency of the study.

In conclusion, our study confirmed the importance of family history as a risk factor for OFCs. Our study did not show an association with folic acid supplementation but was underpowered to detect small differences. The finding of higher risk among vegetarians requires further exploration and research. More studies are needed to establish the role of vitamin B12 alone or in combination with folic acid in prevention of OFCs especially in Indian population where majority are vegetarians.

## Supporting information

Additional Supporting information may be found in the online version of this article.

Supplementary Table S1. Characteristics of the Study Population
