# Supplementary material for: Risk factors for orofacial clefts in India: A case–control study
Source: Birth Defects Res. 2017 Aug 2;109(16):1284–91. doi: 10.1002/bdr2.1073 (PMC6686724; doi:10.1002/bdr2.1073)
Supplement: Supplementary file 1 — Supplementary Table S1. Characteristics of the Study Population [file BDR2-109-1284-s001.docx]

**Tables: Folic acid project - Case control study**

**Web Table 1: Characteristics of the study population**

| **Variables** | **Overall n=785** | **Delhi**  **n=260** | **Hyderabad**  **n=250** | **Bengaluru**  **n=275** |
| --- | --- | --- | --- | --- |
| **Age of child in days** mean (SD) [Range] | 23.9 (36.7)  [0, 120] | 20.3 (35.1) [0, 120] | 19.5 (34.7)  [0, 120] | 31.2 (38.8) [0, 120] |
| **Sex of child**  -Male  -Female | 424 (54.0)  361 (46.0) | 139 (53.5)  121 (46.5) | 141 (56.4)  109 (43.6) | 144 (52.4)  131 (47.6) |
| **Parity of mother** [n (%)]  -Primiparous  -Second & third  -Fourth or more | 380 (48.4)  379 (48.3)  26 (3.3) | 116 (44.6)  139 (53.5)  5 (1.9) | 120 (48.0)  110 (44.0)  20 (8.0) | 144 (52.4)  130 (47.3)  1 (0.4) |
| **Age of mother (in completed years)** mean (SD) [Range] | 24.2 (3.5)  [18, 38] | 23.4 (2.9) [18, 33] | 25.2 (3.8) [18, 38] | 24.0 (3.7)  [18, 38] |
| **Age of Father (in completed years)** [mean (SD)] | 29.0 (4.3)  [20, 48] | 28.4 (4.1)  [20, 45] | 29.0 (4.5)  [22, 48] | 29.6 (4.3)  [21, 45] |
| **Education status of mother** [n (%)]  **-**Illiterate  **-**Can read & Write but no formal education  **-**Primary School  -High & Senior secondary  **-**Graduation & above | 75 (9.5)  21 (2.7)  52 (6.6)  507 (64.6)  130 (16.6) | 30 (11.5)  4 (1.5)  17 (6.5)  161 (61.9)  48 (18.5) | 37 (14.8)  3 (1.2)  23 (9.2)  139 (55.6)  48 (19.2) | 8 (2.9)  14 (5.1)  12 (4.4)  207 (75.3)  34 (12.4) |
| **Education status of father** [n (%)]  **-**Illiterate  **-**Can read & Write but no formal education  **-**Primary School  -High & Senior secondary  **-**Graduation & above | 77 (9.8)  27 (3.4)  55 (7.0)  466 (59.4)  160 (20.4) | 41 (15.8)  7 (2.7)  16 (6.1)  141 (54.2)  55 (21.1) | 21 (8.4)  4 (1.6)  13 (5.2)  149 (59.6)  63 (25.2) | 15 (5.4)  16 (5.8)  26 (9.4)  176 (64.0)  42 (15.3) |
| **Caste** [n (%)]  **-**SC  -ST  -BC  -OC  -Don’t know | 142 (18.1)  51 (6.5)  259 (33.0)  311 (39.6)  22 (2.8) | 70 (26.9)  16 (6.1)  113 (43.5)  55 (21.1)  6 (2.3) | 33 (13.2)  9 (3.6)  20 (9.80)  179 (71.6)  9 (3.6) | 39 (14.2)  26 (9.4)  126 (45.8)  77 (28.0)  7 (2.5) |
| **Occupation of mother** [n (%)]  **-**Homemaker  -Agriculture  -Office Job  -Industry  -Manual Labour  -Business  -Others | 676 (86.1)  28 (3.6) 15 (1.9)  9 (1.1) 24 (5.0)  6 (0.7)  27 (3.4) | 199 (76.5)  22 (8.5)  5 (1.9)  1 (0.3)  13 (5.0)  2 (0.7)  18 (6.9) | 237 (94.8)  1 (0.4)  0  6 (2.4)  3 (1.2)  2 (0.8)  1 (0.4) | 240 (87.3)  5 (1.8)  4 (1.4)  8 (2.9)  8 (2.9)  2 (0.7)  8 (2.9) |
| **Occupation of father** [n (%)]  -Agriculture  -Office Job  -Industry  -Manual Labour  -Business  -no job  -Others | 54 (6.9)  138 (7.6)  48 (6.1)  245 (31.2)  75 (9.5)  10 (1.3)  215 (27.4) | 38 (14.6)  35 (13.5)  7 (2.7)  54 (20.8)  19 (7.3)  6 (2.3)  101 (38.8) | 1 (0.4)  70 (28.0)  11 (4.4)  51 (20.4)  30 (12.0)  3 (1.2)  84 (33.6) | 15 (5.4)  33 (12.0)  30 (10.9)  140 (50.9)  26 (9.4)  1 (0.4)  30 (10.9) |
| **Family Income mean (SD) range**  - <1 lakh [n (%)]  - 1-2 lakh [n (%)]  - Don’t know [n (%)] | 12684.1 (13972.7)  [3000, 160000]  501 (63.8)  3 (0.3)  281 (35.8) | 9330.6 (4529.8) [3000, 30000]  134 (51.5)  0  126 (48.46) | 15236.4 (14724.0) [3000, 80000]  110 (44.00)  0  140 (56.00) | 13332.7 (16382.4) [3000, 160000]  257 (93.4)  3 (0.4)  15 (5.45) |
| **Age at marriage** mean (SD) [range] | 20.2 (3.1) [10, 37] | 19.7 (2.6) [12, 30] | 20.3 (3.4) [10, 29] | 20.6 (3.2) [14, 37] |
| **Marriage within relation** [n (%)]  -Yes  -No | 129 (16.4)  656 (83.6) | 52 (20.0)  208 (80.0) | 12 (4.8)  238 (95.2) | 65 (23.6)  210 (76.4) |
